# Social vulnerability in persons with chronic hepatitis C virus infection is associated with a higher risk of prescription opioid use

**DOI:** 10.1038/s41598-021-85283-6

**Published:** 2021-03-15

**Authors:** Adeel A. Butt, Peng Yan, Shashi Kapadia, Abdul-Badi Abou-Samra, Naveed Z. Janjua, Said Ibrahim

**Affiliations:** 1 VA Pittsburgh Healthcare System, Building 30, Mailstop 151, University Drive C, Pittsburgh, PA 15240 USA; 2 Weill Cornell Medical College, New York, NY USA; 3 Weill Cornell Medical College, Doha, Qatar; 4 Hamad Medical Corporation, Doha, Qatar; 5 University of British Columbia, Vancouver, BC Canada; 6 British Columbia Centre for Disease Control, Vancouver, BC Canada

**Keywords:** Epidemiology, Hepatology, Liver diseases

## Abstract

Prescription opioid use (POU) is often a precursor to opioid use disorder (OUD) and subsequent consequences. Persons with chronic hepatitis C virus infection (CHC) may be at a higher risk of POU due to a higher comorbidity burden and social vulnerability factors. We sought to determine the burden of POU and associated risk factors among persons with CHC in the context of social vulnerability. We identified CHC persons and propensity-score matched HCV− controls in the electronically retrieved Cohort of HCV-Infected Veterans and determined the frequency of acute, episodic long-term and chronic long-term POU and the prevalence of social vulnerability factors among persons with POU. We used logistic regression analysis to determine factors associated with POU. Among 160,856 CHC and 160,856 propensity-score matched HCV-controls, acute POU was recorded in 38.4% and 38.0% (P = 0.01) respectively. Episodic long-term POU was recorded in 3.9% in each group (P = 0.5), while chronic long-term POU was recorded in 28.4% and 19.2% (P < 0.0001). CHC was associated with a higher risk of chronic long-term POU (OR 1.66, 95%CI 1.63, 1.69), but not with acute or episodic long-term POU. Black race, female sex and homelessness were associated with a higher risk of chronic long-term POU. Presence of ≥ 1 factor was associated with a higher risk of all POU patterns. Persons with CHC have more social vulnerability factors and a higher risk of chronic long-term POU. Presence of ≥ 1 social vulnerability factor is associated with a higher risk of POU. Downstream consequences of POU need further study.

## Introduction

Opioid use disorder (OUD) has emerged as a major health threat in the United States^1–6^. Nearly 1% of the population (> 2.5 million persons) suffer from OUD with over 67,367 opioid related deaths reported in 2018^7–9^. Prescription opioid use (POU) has increased in the US in recent years with devastating individual and societal consequences and precedes OUD in a substantial proportion of persons^1,2,10,11^. There is evidence that the growing OUD epidemic is strongly associated with an increase in the incidence of chronic hepatitis C virus (CHC) infection^12–14^. In the US, annual incidence of HCV infection increased from 0.3 to 0.7 cases per 100,000 persons between 2004 and 2014, concurrent with a nearly fourfold increase in treatment admissions for prescription opioid injection use^14^.

Persons with CHC are more likely to have a diagnosis of major psychiatric illness, hazardous alcohol use and drug abuse or dependence^15,16^. The confluence of CHC infection, POU and psychiatric illness poses a unique and specific challenge, exacerbated by the social vulnerability of the persons who are affected by these conditions singly or in any combination thereof. Socially vulnerable populations are groups and communities at a higher risk for poor health, or consequences of illness, as a result of social, cultural, economic, political and environmental disparities. Socially vulnerable populations have been shown to suffer significant disparities in linkage to treatment and clinical outcomes for various conditions^17–23^. Since POU frequently precedes OUD and the consequences associated with OUD, it is important to understand the magnitude and risk factors associated with POU. The temporal association between HCV and POU is complicated and bidirectional, with each being a risk factor for the other. The aim of our current study to determine the burden of POU and associated risk factors among Veterans with CHC infection and matched controls. We did not study the temporal associations of the risk variables in the current study.

## Methods

### Data sources and cohort construction

We used the Electronically Retrieved Cohort of HCV Infected Veterans (ERCHIVES) as the data source for this analysis. ERCHIVES is well defined in numerous previous publications^24–29^. Briefly, all persons with HCV infection in the Department of Veterans Affairs healthcare system (VA) diagnosed after October 1, 2001 are identified based on a positive HCV antibody test. Age (5-year blocks), sex and race matched persons with a negative HCV antibody test in the same year are identified as controls. Demographic, clinical, laboratory, pharmacy, anthropometric, vital signs and survival date are retrieved from the Corporate Data Warehouse (CDW) using well established algorithms and definitions. The cohort is updated annually to identify newly diagnosed cases with HCV infection and corresponding controls.

For the current study, we included all persons in ERCHIVES identified between 2001 and 2019. For persons with HCV, we retained those with at least one detectable HCV RNA, thus fulfilling the criteria for CHC infection. For both groups (those with and without CHC), we excluded those with human immunodeficiency virus coinfection and those with missing data to calculate FIB-4 score within 12 months prior to cohort entry date. A propensity score was calculated for each study participant using a logistic regression model including age, race, sex, body mass index, FIB-4, diabetes, hypertension, coronary artery disease, smoking status and alcohol use disorder. Using the propensity score, we conducted propensity score matching to create 1:1 matched sets of persons with CHC and uninfected controls using a propensity score distance. The tails of the propensity scores were not trimmed. We used the greedy matching (CHC selected at random first, then HCV− whose propensity score is closest to the randomly selected CHC is chosen for matching), matching without replacement (once an HCV− has been selected to be matched, that HCV− is no longer available for selecting), and without restriction for the maximum difference between the propensity scores of matched CHC and HCV−.

### Primary outcome measures

Our primary outcome measures were acute and long-term prescription opioid use (POU). All opioids approved by the US Food and Drug Administration were included when determining POU (list available as Appendix 1 in accompanying supplementary materials). Acute POU was defined as an episode whereby an opioid was prescribed with less than 90 days of drug supply. Long-term POU was defined as at least one episode with ≥ 90 days of drug supply including refills. Long-term POU was further subdivided into episodic and chronic use. Episodic long-term use included the subset of persons receiving ≥ 90 days of POU supply with < 50% episode intensity, where episode intensity was calculated as percentage of episode days covered by the prescription. Chronic long-term use included the subset of persons receiving ≥ 90 days of POU supply with > 50% episode intensity.

### Clinical covariates

Diabetes was defined using a combination of blood glucose measurements, prescription of insulin or oral hypoglycemic and ICD-9/10 codes^27,30^. We used FIB-4 to estimate the severity of liver disease and presence of cirrhosis. FIB-4 score was calculated based on clinical and an average of two lab values recorded within 12 months preceding entry into the cohort^31^. Psychiatric illness (major depression, schizophrenia, bipolar disorder, post-traumatic stress disorder), alcohol use disorder, hypertension, cancer diagnosis and cardiovascular disease were defined by presence of at least one inpatient or two outpatient International Classification of Diseases, 9th edition, Clinical Modification (ICD-9CM, prior to October 1, 2015) or ICD-10 (from October 1, 2015 onwards) codes^32^. Smoking history was retrieved from the Health Factors Dataset and classified as never, former and current smokers^33^. Since opioid analgesics are often prescribed for short term pain relief after dental procedures and fractures, we identified persons who underwent removal or restoration of teeth and those who sustained major joint dislocation or bone fracture using ICD-9CM and ICD-10 codes.

### Social vulnerability factors

For our current study, we identified the following factors to denote social vulnerability: (1) non-White race; (2) female sex; (3) homelessness; (4) poverty, defined as annual income less than $45,200 thus placing them in the low income or below Federal poverty level; (5) rural or highly rural residence; (6) alcohol use disorder; and (7) diagnosis of psychiatric illness. These variables were retrieved from the CDW datasets where they are recorded based on VA’s established algorithms. These factors were chosen to represent persons with social vulnerability since persons with these characteristics have been shown to suffer significant disparities in linkage to treatment and clinical outcomes for various conditions^17–23^.

### Statistical analyses

Baseline characteristics of persons with CHC and uninfected controls, and persons with and without POU were compared using chi-squared test for dichotomous variables and *t-test* for continuous variables. We determined the proportion of persons with acute, episodic long-term and chronic long-term POU by number of social vulnerability factors. We also determined the number of social vulnerability factors present among those with CHC only, CHC with POU and POU without CHC. We used logistic regression to generate odds ratios and 95% confidence intervals for factors associated with acute or long-term POU. Covariates of interest were demographic and clinical characteristics at baseline. All statistical analyses were completed using SAS Version 9.4 (SAS Institute Inc., Cary NC).

### Missing data

There were 1707 (0.5%) participants with missing income data and 446 (0.1%) with missing data on their residence. These persons were not excluded form the main analysis since they comprised a very small proportion of the study population. We compared the baseline characteristics of persons with and without CHC, and with and without POU to ensure that the overall characteristics were not different in the analysis cohort.

### Ethical approval

The study was approved by the Institutional Review Board at VA Pittsburgh Healthcare System and all methods were carried out in accordance with relevant guidelines and regulations. A waiver of informed consent requirement was granted to studies related to ERCHIVES by the same Board.

## Results

### Baseline characteristics: CHC vs HCV−

**Figure 1:**
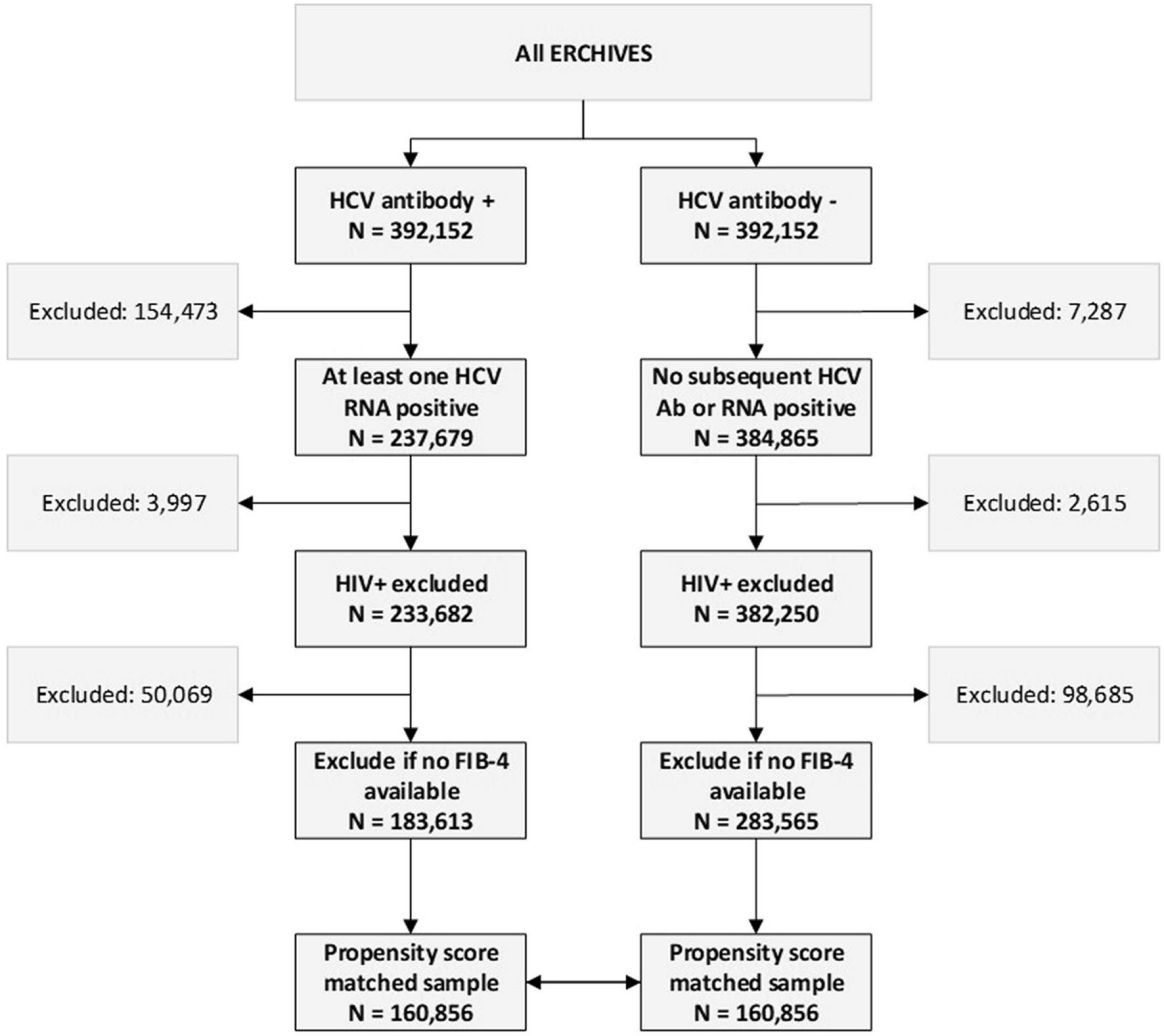
Cohort construction.

Our final dataset included 160,856 persons with CHC and 160,856 propensity score matched controls without CHC infection (Fig. 1). Baseline characteristics of persons with CHC and uninfected controls are shown in Table 1. Median age was 54 (IQR 49, 59) years for those with CHC and 53 (IQR 48, 59) years for HCV uninfected controls. Ninety-six per cent were males and 54% were White. Persons with CHC were more likely to be homeless (21.7% vs. 11.2%; P < 0.0001), reside in an urban area (73.3% vs. 67.9%; P < 0.0001) and in the low income or below federal poverty level group (94.5% vs. 89.7%; P < 0.0001). Persons with CHC infection were also more likely to have a psychiatric diagnosis (32.1% vs. 25.6%; P < 0.0001).

**Table 1:** Baseline characteristics of persons with CHC and propensity score matched HCV-uninfected controls.

	CHC N = 160,856	HCV− N = 160,856	P-value
Age, median (IQR)	54 (49,59)	53 (48,59)	< 0.0001
**Race**			< 0.0001
White	54.0%	54.3%	
Black	30.0%	29.2%	
Hispanic	3.5%	3.6%	
Others/unknown	12.5%	12.9%	
Male sex	96.6%	96.4%	0.0003
Diabetes	5.9%	5.1%	< 0.0001
Hypertension	44.5%	43.7%	< 0.0001
Cardiovascular disease	10.1%	9.9%	0.08
Cancer diagnosis	6.4%	5.2%	< 0.0001
Alcohol use disorder	36.6%	26.0%	< 0.0001
**Smoking**			< 0.0001
Former	53.0%	51.6%	
Never	13.7%	14.4%	
Current	11.6%	9.3%	
Unknown	21.7%	24.7%	
Homeless	21.7%	11.2%	< 0.0001
**Residence**			< 0.0001
Highly rural	0.9%	1.2%	
Rural	25.8%	31.0%	
Urban	73.3%	67.9%	
**Income level**			< 0.0001
< 21,330 (below Federal poverty level)	71.5%	60.8%	
21,330–45,200 (low income)	22.9%	28.9%	
45,201–135,600 (middle class)	5.3%	9.7%	
> 135,600 (high income)	0.2%	0.6%	
**Any psychiatric diagnosis**	32.1%	25.6%	< 0.0001
Major depression	15.3%	11.2%	< 0.0001
Schizophrenia	6.1%	4.6%	< 0.0001
Bipolar disorder	9.0%	5.5%	< 0.0001
Post-traumatic stress disorder	17.1%	13.5%	< 0.0001
Acute prescription opioid use	38.4%	37.9%	0.01
**Long-term prescription opioid use**	32.3%	23.1%	< 0.0001
Episodic long-term	3.9%	3.9%	0.54
Chronic long-term	28.4%	19.2%	< 0.0001
Three or more social vulnerability factors	48.8%	39.5%	< 0.0001

HCV, hepatitis C virus; CHC, chronic hepatitis C virus infection.

Acute POU was numerically similar in both groups (38.4% vs. 37.9%; P = 0.01); however long-term POU was higher among persons with CHC (32.3% vs. 23.1%) driven entirely by chronic long-term use (28.4% vs. 19.2%) with no difference in episodic long-term use (3.9% vs. 3.9%). Proportion of persons with 3 or more social vulnerability factors was higher among persons with CHC (48.8% vs. 39.5%).

We compared the baseline characteristics of persons with and without CHC after excluding those with missing data to ensure that the overall characteristics were not different in the analysis cohort. The results were nearly identical to the main analysis results (Supplementary Table 1).

### Baseline characteristics: POU+ vs POU−

Persons with any POU (acute or long-term) were more likely to have comorbid conditions including diabetes (6.6% vs. 3.4%), hypertension (47.0% vs. 38.6%), cardiovascular disease (11.4% vs. 7.2%), cancer diagnosis (7.0% vs. 3.5%) and alcohol use disorder (34.6% vs. 24.9%) compared with those with no POU (P < 0.0001 for all comparisons) (Table 2). They were also more likely to be homeless (18.3% vs. 12.9%), have lower income level (67.3% vs. 64.1% below the Federal poverty line) and a diagnosis of psychiatric illness (32.5% vs. 21.8%) (P < 0.0001 for all comparisons).

**Table 2:** Baseline characteristics of persons with and without any acute or chronic prescription opioid use.

	POU+ N = 211,799	POU− N = 109,913	P-value
Age, median (IQR)	53 (48, 58)	55 (49, 61)	< 0.0001
**Race, %**			< 0.0001
White	54.6%	53.3%	
Black	31.2%	26.5%	
Hispanic	3.8%	3.2%	
Others/unknown	10.5%	17.0%	
Sex, % male	96.4%	96.7%	0.0002
Diabetes, %	6.6%	3.4%	< 0.0001
Hypertension, %	47.0%	38.6%	< 0.0001
Cardiovascular disease, %	11.4%	7.2%	< 0.0001
Cancer diagnosis, %	7.0%	3.5%	< 0.0001
Alcohol use disorder, %	34.6%	24.9%	< 0.0001
**Smoking, %**			< 0.0001
Current	53.9%	49.2%	
Former	12.8%	16.4%	
Never	9.8%	11.7%	
Unknown	23.4%	22.7%	
Homeless	18.3%	12.9%	< 0.0001
**Residence**			< 0.0001
Highly rural	1.0%	1.1%	
Rural	28.0%	29.2%	
Urban	71.0%	69.8%	
**Income level**			< 0.0001
< 21,330 (below Federal poverty level)	67.3%	64.1%	
21,330–45,200 (low income)	26.8%	24.3%	
45,201–135,600 (middle class)	5.8%	10.8%	
> 135,600 (high income)	0.2%	0.9%	
**Any psychiatric diagnosis, %**	32.5%	21.8%	< 0.0001
Major depression, %	15.6%	8.8%	< 0.0001
Schizophrenia, %	5.6%	4.8%	< 0.0001
Bipolar disorder, %	8.3%	5.0%	< 0.0001
Post-traumatic stress disorder, %	17.7%	10.7%	< 0.0001

POU, prescription opioid use.

We compared the baseline characteristics of persons with and without POU after excluding those with missing data to ensure that the overall characteristics were not different in the analysis cohort. The results were nearly identical to the main analysis results (Supplementary Table 2).

### POU by social vulnerability factors

Proportion of persons with POU increased with increasing number of social vulnerability factors (Table 3). Among persons with acute POU, 29.46% had no social vulnerability factors, while among those with long-term episodic POU, 2.32% had no social vulnerability factor, and among those with long-term chronic POU, 11.99% had no social vulnerability factor. These proportions were higher for persons with any social vulnerability factor. However, there was no discernible relationship with increasing number of social vulnerability factors and higher rates of POU (Table 3).

**Table 3:** Prescription opioid use by number of social vulnerability factors (non-white race, females, homelessness, poverty income < $45,200, rural or highly rural residence, alcohol use disorder, psychiatric illness).

	N	Acute POU, %	Long-term episodic POU, %	Long-term chronic POU, %
No factor	6080	29.5	2.3	12.0
Any one factor	315,632	38.3	3.9	24.0
Any two factors	258,280	39.0	4.1	24.7
Any three factors	142,124	40.5	4.2	26.3
Any four factors	60,785	42.7	4.2	27.7
Any five factors	17,344	44.0	4.5	28.4
Any six factors	1759	42.7	4.7	30.5
All seven factors	28	35.7	3.6	25.0

POU, prescription opioid use.

### Factors associated with POU

We determined the factors associated with acute, episodic long-term and chronic long-term POU. Increasing age was associated with lower risk of all types of POU. Black and Hispanic races were associated with a higher risk of acute and episodic long-term POU but with a lower risk of chronic long-term use (Table 4). Medical comorbidities (diabetes, cardiovascular disease, cancer) were associated with a higher risk of all types of POU. Rural and highly rural residence were associated with a lower risk of acute POU, but a higher risk of chronic long-term POU. Higher income strata were associated with a lower risk of all types of POU. CHC infection was not associated with episodic long-term POU (OR 0.99, 95% CI 0.95, 1.03), but was significantly associated with chronic long-term POU (OR 1.66, 95% CI 1.63, 1.69) (Table 4). Presence of any social vulnerability factor was associated with a higher risk of acute, episodic long-term and chronic long-term POU, with increasing number of factors associated with a generally higher risk, though the relationship was not clearly linear (Supplementary Table 3).

**Table 4:** Predictors of prescription opioid use (multivariable logistic regression model).

	Acute POU		Long-term episodic POU		Long-term chronic POU	
	Odds ratio	95% CI	Odds ratio	95% CI	Odds ratio	95% CI
Age, per 10-year increase	0.92	0.91, 0.92	0.91	0.89,0.93	0.79	0.78,0.80
**Race (comparator: white)**						
Black	1.34	1.32, 1.36	1.29	1.23, 1.34	0.71	0.70, 0.73
Hispanic	1.50	1.44, 1.55	1.22	1.11, 1.34	0.66	0.62, 0.69
Others/unknown	0.92	0.9, 0.94	0.88	0.82, 0.93	0.66	0.65, 0.68
Male sex (vs. female)	0.96	0.92, 1.00	0.81	0.74, 0.89	1.14	1.09, 1.19
Diabetes	1.16	1.13, 1.20	1.36	1.27, 1.45	1.24	1.19, 1.28
Hypertension	1.01	0.99, 1.02	1.32	1.26, 1.37	1.48	1.45, 1.50
Cardiovascular disease	1.11	1.08, 1.13	1.37	1.30, 1.45	1.25	1.22, 1.29
Cancer diagnosis	1.22	1.19, 1.26	1.52	1.43, 1.63	1.68	1.63, 1.74
Alcohol use disorder	1.17	1.15, 1.19	0.94	0.90, 0.99	0.98	0.96, 1.00
**Smoking (comparator: never)**						
Former	0.96	0.93, 0.98	0.99	0.92, 1.06	1.09	1.05, 1.13
Current	0.94	0.91, 0.96	0.96	0.90, 1.02	1.45	1.41, 1.50
Unknown	0.98	0.96, 1.01	1.07	1.00, 1.14	1.38	1.34, 1.43
Homeless (vs. not homeless)	1.12	1.09, 1.14	0.91	0.86, 0.96	0.92	0.90, 0.95
**Residence (vs. urban residence)**						
Rural residence	0.83	0.82, 0.85	1.05	1.01, 1.09	1.27	1.25, 1.30
Highly rural residence	0.82	0.76, 0.88	0.99	0.82, 1.20	1.37	1.27, 1.49
**Income level (vs. < 21,330)**						
21,330–45,200 (low income)	1.09	1.07, 1.11	1.17	1.12, 1.22	0.98	0.96, 0.99
45,201–135,600 (middle class)	0.92	0.90, 0.95	0.89	0.82, 0.96	0.56	0.54, 0.59
> 135,600 (high income)	0.5	0.44, 0.58	0.51	0.34, 0.77	0.23	0.18, 0.29
Psychiatric illness	1.00	0.98, 1.01	1.19	1.14, 1.24	1.39	1.36, 1.41
CHC	0.98	0.96, 0.99	0.99	0.95, 1.03	1.66	1.63, 1.69

CHC, chronic hepatitis C virus infection; POU, prescription opioid use.

## Discussion

We provide the first comprehensive picture of POU in persons with CHC and appropriately matched controls in the context of social vulnerability. We found that CHC and multiple social vulnerability factors are associated with a higher risk of chronic long-term POU, but not necessarily acute POU.

We found that CHC was associated with a significantly higher risk of chronic long-term POU but not with a higher risk of acute or episodic long-term POU. In fact, CHC was among the strongest risk factor for POU with the odds ratio similar to cancer diagnosis. CHC is associated with a higher burden of medical and psychiatric comorbidities and several extrahepatic manifestations^34,35^. Chronic pain is often associated with CHC infection itself, or with one of several rheumatologic or immunologic manifestations of CHC infection, with up to two-thirds of the patients with CHC experiencing chronic pain^35,36^. In the interferon-era, treatment itself was associated with chronic fatigue and pain. However, since only a small fraction of persons were ever treated with interferon-based regimens, other factors certainly contributed to chronic pain^37^. Newer all-oral direct acting antiviral agents are more tolerable, more efficacious and have fewer side effects. Whether treatment with these newer agents is associated with a lower incidence of pain or resolution of chronic pain requires further study. Such positive association may in turn reduce the need for opioid prescriptions and its downstream consequences. On the other hand, the association between CHC and POU could be due to POU leading to injection drug use, which increases the risk of acquiring CHC. Future studies should also determine the direction of association between CHC and POU.

Persons with POU were more likely to have medical comorbidities. While most comorbidities studied do not necessarily require opioids, they may be a surrogate for other associated conditions. We also found POU to be more common among persons with several individual social vulnerability factors. These included Black race, homelessness, lower income strata and psychiatric illness. Rural or highly rural residence was not associated with a higher rate of POU. However, in logistic regression model, male sex was associated with a lower risk of acute or episodic long-term POU but a higher risk of chronic long-term POU. Whether females have actually lesser need for chronic long-term opioids, or whether this is due to lesser access to resources or perception of providers is unknown and warrants further study. Risk of POU was significantly lower in persons with higher income strata independent of other factors studied confirming that persons in lower economic strata are most hardly hit by POU, and by extension possibly opioid use disorder. This highlights co-occurrence of syndemics of socioeconomic marginalization and other vulnerability factors with POU use, which could be related to eventual opioid use disorders.

These findings, taken together, indicate a strong association between POU and socially vulnerable CHC infected persons. Since opioid analgesics are frequently prescribed for appropriate medical conditions, an important next step is to determine the proportion of persons using opioid analgesics strictly as prescribed, and to determine the magnitude and factors associated with inappropriate use. Subsequent studies should also determine the proportion of persons with POU progressing to OUD and injection drug use, and risk factors associated with such progression.

Strengths of our study include a large national population and appropriately matched controls. Veterans have a high burden of medical, psychiatric and substance use comorbidities. While this is not truly representative of the overall population, it is critical to understand the magnitude and impact of this epidemic in this particularly high-risk population. A main limitation of the current study is that we only studied POU and not the downstream consequences, namely OUD and illicit injection drug use. However, our specific aim for this study was limited to understanding the first step in this cascade and future studies will look into these downstream effects and clinical consequences. Another limitation of our study is the absence of a quantitative measure of pain which may require opioid prescription. To mitigate this at least partially, we excluded persons with common conditions associated with short-term POU, including acute fractures and dental work, as well as chronic painful conditions like cancer. Our study does not address the question of temporal relationships between POU, injection drug use and CHC infection, since this was not the aim of our study.

In conclusion, persons with CHC have high prevalence of social vulnerability factors, and a higher risk of chronic long-term POU. Consequences of high rate and risk of POU among this population need further study.

## Supplementary Information

Supplementary Information.
