# Supplementary material for: Social vulnerability in persons with chronic hepatitis C virus infection is associated with a higher risk of prescription opioid use
Source: Sci Rep. 2021 Mar 15;11:5883. doi: 10.1038/s41598-021-85283-6 (PMC7961056; doi:10.1038/s41598-021-85283-6)
Supplement: Supplementary file 1 — Supplementary Information. [file 41598_2021_85283_MOESM1_ESM.docx]

**Social Vulnerability in Persons with Chronic Hepatitis C Virus Infection is Associated with a Higher Risk of Prescription Opioid Use**

Short running title: Prescription opioid use in HCV

Adeel A. Butt, MBBS, MS^1,2,3,4^ aab2005@qatar-med.cornell.edu

Peng Yan, MD, MS^1^ peng.yan@va.gov

Shashi Kapadia, MD^2^ shk9078@med.cornell.edu

Abdul-Badi Abou-Samra, MD, PhD^4^ asamra@hamad.qa

Naveed Z. Janjua, MBBS, DrPH^5,6^ naveed.janjua@bccdc.ca

Said Ibrahim, MD, MPH^2^ sai2009@med.cornell.edu

1 VA Pittsburgh Healthcare System, Pittsburgh, PA, USA

2 Weill Cornell Medical College, New York, NY, USA

3 Weill Cornell Medical College, Doha, Qatar

4 Hamad Medical Corporation, Doha, Qatar

5 University of British Columbia, Vancouver, BC, Canada
6 British Columbia Centre for Disease Control

Supplementary table 1. Baseline characteristics of persons with and without hepatitis C virus infection after excluding those with missing data.

|  | HCV+  N=159755 | HCV-  N=159806 | P-value |
| --- | --- | --- | --- |
| Age, median (IQR) | 54 (49,59) | 53 (48,59) | <.0001 |
| Race, % |  |  | <.0001 |
| White | 54.00% | 54.28% |  |
| Black | 30.03% | 29.29% |  |
| Hispanic | 3.51% | 3.63% |  |
| Others/unknown | 12.46% | 12.80% |  |
| Sex, % male | 96.64% | 96.40% | 0.0003 |
| Diabetes, % | 5.93% | 5.17% | <.0001 |
| Hypertension, % | 44.77% | 43.91% | <.0001 |
| Cardiovascular disease, % | 10.13% | 9.96% | 0.11 |
| Cancer diagnosis, % | 6.41% | 5.26% | <.0001 |
| Alcohol use disorder, % | 36.68% | 26.12% | <.0001 |
| Smoking, % |  |  | <.0001 |
| Current | 53.02% | 51.62% |  |
| Former | 13.66% | 14.39% |  |
| Never | 11.60% | 9.27% |  |
| Unknown | 21.71% | 24.72% |  |
| Homeless | 21.74% | 11.21% | <.0001 |
| Residence |  |  | <.0001 |
| Highly rural | 0.86% | 1.18% |  |
| Rural | 25.81% | 30.96% |  |
| Urban | 73.33% | 67.86% |  |
| Income level |  |  | <.0001 |
| <21,330 (below Federal poverty level) | 71.36% | 60.64% |  |
| 21,330 – 45,200 (low income) | 23.08% | 29.01% |  |
| 45,201 – 135,600 (middle class) | 5.33% | 9.73% |  |
| >135,600 (high income) | 0.23% | 0.62% |  |
| Any psychiatric diagnosis, % | 32.15% | 25.59% | <.0001 |
| Major depression, % | 15.28% | 11.23% | <.0001 |
| Schizophrenia, % | 6.08% | 4.67% | <.0001 |
| Bipolar disorder, % | 8.97% | 5.50% | <.0001 |
| PTSD, % | 17.13% | 13.51% | <.0001 |
| Acute prescription opioid use | 38.47% | 38.05% | 0.01 |
| Long-term prescription opioid use | 32.43% | 23.21% | <.0001 |
| Episodic long-term | 3.92% | 3.96% | 0.61 |
| Chronic long-term | 28.51% | 19.25% | <.0001 |
| Three or more social vulnerability factors | 48.86% | 39.61% | <.0001 |

Supplementary table 2. Baseline characteristics of persons with and without prescription opioid use after excluding those with missing data.

|  | POU+  N=211179 | POU-  N=108382 | P-value |
| --- | --- | --- | --- |
| Age, median (IQR) | 53 (48,58) | 55 (49,61) | <.0001 |
| Race, % |  |  | <.0001 |
| White | 54.58% | 53.28% |  |
| Black | 31.22% | 26.62% |  |
| Hispanic | 3.76% | 3.19% |  |
| Others/unknown | 10.43% | 16.91% |  |
| Sex, % male | 96.42% | 96.70% | <.0001 |
| Diabetes, % | 6.63% | 3.44% | <.0001 |
| Hypertension, % | 47.08% | 39.00% | <.0001 |
| Cardiovascular disease, % | 11.45% | 7.30% | <.0001 |
| Cancer diagnosis, % | 7.04% | 3.49% | <.0001 |
| Alcohol use disorder, % | 34.64% | 25.09% | <.0001 |
| Smoking, % |  |  | <.0001 |
| Current | 53.90% | 49.25% |  |
| Former | 12.84% | 16.34% |  |
| Never | 9.79% | 11.70% |  |
| Unknown | 23.47% | 22.72% |  |
| Homeless | 18.30% | 12.92% | <.0001 |
| Residence |  |  | <.0001 |
| Highly rural | 1.00% | 1.06% |  |
| Rural | 28.00% | 29.13% |  |
| Urban | 71.00% | 69.81% |  |
| Income level |  |  | <.0001 |
| <21,330 (below Federal poverty level) | 67.20% | 63.66% |  |
| 21,330 – 45,200 (low income) | 26.82% | 24.54% |  |
| 45,201 – 135,600 (middle class) | 5.81% | 10.89% |  |
| >135,600 (high income) | 0.18% | 0.91% |  |
| Any psychiatric diagnosis, % | 32.49% | 21.82% | <.0001 |
| Major depression, % | 15.60% | 8.68% | <.0001 |
| Schizophrenia, % | 5.66% | 4.82% | <.0001 |
| Bipolar disorder, % | 8.36% | 5.05% | <.0001 |
| PTSD, % | 17.69% | 10.70% | <.0001 |
| Fracture diagnosis +/- 14 days of POU | 6.29% | 0.00% | <.0001 |
| Dental procedure | 0.01% | 0.00% | 0.27 |

Supplementary table 3. Predictors of prescription opioid use including number of social vulnerability factors (Non-White race, Females, Homelessness, Poverty income <$45,200, Rural or highly rural residence, Alcohol use disorder, psychiatric illness) (logistic regression model).

|  | Acute POU |  | Long-term episodic POU |  | Long-term chronic POU |  |
| --- | --- | --- | --- | --- | --- | --- |
|  | Odds ratio | 95% CI | Odds ratio | 95% CI | Odds ratio | 95% CI |
| Age, per 10-year increase | 0.91 | 0.9,0.92 | 0.91 | 0.89,0.93 | 0.78 | 0.77,0.79 |
| Diabetes | 1.2 | 1.16,1.23 | 1.39 | 1.3,1.49 | 1.23 | 1.19,1.28 |
| Hypertension | 1.04 | 1.03,1.06 | 1.35 | 1.3,1.41 | 1.43 | 1.41,1.46 |
| Cardiovascular disease | 1.11 | 1.09,1.14 | 1.37 | 1.3,1.45 | 1.3 | 1.26,1.33 |
| Cancer diagnosis | 1.19 | 1.15,1.23 | 1.51 | 1.41,1.61 | 1.78 | 1.72,1.84 |
| Social vulnerability factors (comparator: 0) |  |  |  |  |  |  |
| 1 factors | 1.28 | 1.21,1.36 | 1.45 | 1.22,1.73 | 1.71 | 1.58,1.86 |
| 2 factors | 1.38 | 1.31,1.46 | 1.62 | 1.37,1.92 | 1.79 | 1.65,1.93 |
| 3 factors | 1.46 | 1.38,1.55 | 1.74 | 1.47,2.07 | 1.94 | 1.79,2.1 |
| 4 factors | 1.66 | 1.57,1.76 | 1.69 | 1.42,2.01 | 1.99 | 1.84,2.16 |
| 5 factors | 1.79 | 1.68,1.91 | 1.83 | 1.52,2.2 | 1.93 | 1.77,2.1 |
| 6 factors | 1.67 | 1.5,1.87 | 1.86 | 1.4,2.46 | 2.05 | 1.8,2.34 |
| All seven factors | 1.22 | 0.56,2.65 | 1.53 | 0.21,11.37 | 1.46 | 0.61,3.48 |
| Smoking  (Comparator: Never) |  |  |  |  |  |  |
| Former | 0.94 | 0.91,0.97 | 0.98 | 0.92,1.06 | 1.12 | 1.08,1.17 |
| Current | 0.92 | 0.9,0.95 | 0.94 | 0.88,0.99 | 1.53 | 1.48,1.58 |
| Unknown | 0.99 | 0.96,1.02 | 1.06 | 1,1.14 | 1.4 | 1.36,1.45 |
| HCV+ | 0.99 | 0.98,1.01 | 0.97 | 0.93,1 | 1.66 | 1.63,1.69 |

Appendix 1. List of Opioids.

| Abstral, Actiq (fentanyl) |
| --- |
| Anexsia (hydrocodone/acetaminophen) |
| Avinza (morphine sulfate) |
| Buprenorphine (Butrans) |
| Co-Gesic (hydrocodone/acetaminophen) |
| Demerol |
| Dilaudid (hydromorphone) |
| Duragesic (fentanyl) |
| Embeda (morphine/naltrexone) * |
| Exalgo (hydromorphone hydrochloride) |
| Fentanyl |
| Fentanyl transdermal system |
| Fentora (fentanyl) |
| Hycet (hydrocodone/acetaminophen) |
| Hycodan (hydrocodone/homatropine) |
| Hydromet (hydrocodone/homatropine) |
| Hydromorphone hydrochloride extended-release capsules |
| Hydromorphone hydrochloride extended-release tablets |
| Hysingla (hyrocodone) |
| Ibudone (hydrocodone/ibuprofen) |
| Kadian (morphine sulfate) |
| Liquicet (hydrocodone/acetaminophen) |
| Lorcet (hydrocodone/acetaminophen) |
| Lortab (hydrocodone/acetaminophen) |
| Maxidone (hydrocodone/acetaminophen) |
| Methadone Hydrochloride |
| Morphabond (morphine) |
| Morphine |
| Morphine sulfate and naltrexone extended-release capsules |
| Morphine sulfate controlled-release tablets |
| Morphine sulfate extended-release capsules |
| Morphine sulfate extended-release capsules |
| Norco (hydrocodone/acetaminophen) |
| Nucynta ER (tapentadol) |
| Onsolis (fentanyl) |
| Oramorph (morphine) |
| Oxaydo (oxycodone) |
| Oxycet (oxycodone/acetaminophen) |
| Oxycodone hydrochloride controlled-release tablets |
| OxyContin (oxycodone hydrochloride) |
| Oxymorphone |
| Oxymorphone hydrochloride extended-release tablets |
| Palladone (hydromorphone hydrochloride) ** |
| Percocet (oxycodone/acetaminophen) |
| Percodan (oxycodone/aspirin) |
| Reprexain (hydrocodone/ibuprofen) |
| Rezira (hydrocodone/pseudoephedrine) |
| Roxanol-T (morphine) |
| Roxicet (oxycodone/acetaminophen) |
| Sublimaze (fentanyl) |
| Tapentadol extended-release oral tablets |
| Targiniq ER (oxycodone/naloxone) |
| TussiCaps and Tussionex (hydrocodone/chlorpheniramine) |
| Tylenol #3 and #4 (codeine/acetaminophen) |
| Vicodin (hydrocodone/acetaminophen) |
| Vicoprofen (hydrocodone/ibuprofen) |
| Vituz (hydrocodone/chlorpheniramine) |
| Xartemis XR ( oxycodone/acetaminophen) |
| Xodol (hydrocodone/acetaminophen) |
| Xtampza ER (oxycodone) |
| Zohydro ER (hydrocodone) |
| Zolvit (hydrocodone/acetaminophen) |
| Zutripro (hydrocodone/chlorpheniramine/pseudoephedrine) |
| Zydone (hydrocodone/acetaminophen) |

*Not currently available or marketed due to a voluntary recall but is still approved.

**No longer being marketed but is still approved.
